# Fragment size and dynamics of *EGFR*-mutated tumor-derived DNA provide prognostic information regarding EGFR-TKI efficacy in patients with *EGFR*-mutated NSCLC

**DOI:** 10.1038/s41598-022-17848-y

**Published:** 2022-08-08

**Authors:** Kei Kunimasa, Kazumi Nishino, Yoshiharu Sato, Masahide Mori, Shoichi Ihara, Hidekazu Suzuki, Izumi Nagatomo, Toru Kumagai, Toshitaka Morishima, Fumio Imamura

**Affiliations:** 1 Department of Thoracic Oncology, Osaka International Cancer Institute, 3-1-69 Otemae Chuoku, Osaka City, Osaka, 541-8567 Japan; 2 DNA Chip Research Inc, Tokyo, Japan; 3 Department of Thoracic Oncology, Osaka Toneyama Medical Center, Osaka, Japan; 4 Department of Respiratory Medicine, Osaka Police Hospital, Osaka, Japan; 5 Department of Thoracic Oncology, Osaka Habikino Medical Center, Osaka, Japan; 6 Department of Respiratory Medicine and Clinical Immunology, Graduate School of Medicine, Osaka University, Osaka, Japan; 7 Cancer Control Center, Osaka International Cancer Institute, Osaka, Japan

**Keywords:** Biomarkers, Medical research, Oncology

## Abstract

Circulating tumor DNA (ctDNA)-based next-generation sequencing (NGS) is a complementary and alternative test to tissue-based NGS. We performed NGS analysis of ctDNA samples collected from patients with *EGFR*-mutated non-small cell lung cancer (NSCLC) who received osimertinib; the samples were collected after second-line treatment, before osimertinib treatment, one week and one month after osimertinib treatment, and at the time of resistance formation. We examinedthe correlation with osimertinib efficacy. From January to December 2018, 34 patients with *EGFR*-mutated NSCLC harboring *EGFR* T790M mutations were enrolled, and a total of 132 peripheral blood samples were collected. The fragment sizes of *EGFR*-mutated ctDNAs were significantly shorter than that of their corresponding normal fragments. Osimertinib treatment of patients with shorter *EGFR*-mutated ctDNA fragments resulted in shorter progression-free survival (PFS). The disappearance time of *EGFR*-mutated fragment fractions and clonal evolution patterns (new driver mutation group, additional mutation group vs. attenuation group) were each associated with the PFS achieved with osimertinib treatment; however,multivariate analysis revealed that only shorter *EGFR*-mutated ctDNA fragments were associated with the PFS resulting from osimertinib treatment. *EGFR*-mutated ctDNA fragment size, time of disappearance of these fragments, and clonal evolution pattern were related to the effects of osimertinib. In particular, short *EGFR*-mutated ctDNA fragmentation may be closely related to osimertinib efficacy prediction.

## Introduction

In solid tumors, including lung cancer, the guidelines recommend identification of driver gene mutations and treatment with the corresponding molecular targeted therapies, because of their efficacy, when systemic chemotherapy is introduced^1,2^. In lung cancer, advancements have been made in the identification of druggable driver mutations, and detection of multiple druggable driver mutations before treatment is essential^1,2^. To identify these mutations, next-generation sequencing (NGS) is being rapidly implemented in clinical practice^3^. However, unlike other cancer types, lung cancer requires successful NGS with the relatively small specimens obtainable from bronchoscopic biopsies, especially in advanced cases, and it may not be possible to secure sufficient tumor tissue specimens for successful NGS analysis^4^. In such situations, liquid biopsy can be an extremely powerful tool in lung cancer treatment because it does not require tissue sample; in liquid biopsies, NGS analysis is based on evaluation of cell-free DNA (cfDNA) or circulating tumor-derived DNA (ctDNA) using peripheral blood plasma^5–7^.

Research on liquid biopsy for detecting driver mutations in primary tumors by ctDNA analysis has been accelerating in recent years^7^. NGS panel analysis based on ctDNA is expected to be implemented soon in clinical practice^8,9^. However, NGS analysis of ctDNA that is not seen in DNA from tissues may have limitations. ctDNA is fragmented at lower concentrations than DNA from tissues; therefore, the variant allele frequency (VAF) detected tends to be lower, and the possibility of false-positive or false-negative results associated with this approach should be considered^10^. To address these problems, methods have been developed for increasing the sensitivity of NGS analysis of ctDNA^11^. Barcode sequences with lengths of 10–15 base pairs are added to each ctDNA fragment for distinguishing the sequence read of each fragment^12,13^. However, the disadvantage of this method is that errors in the barcode sequence affect the sensitivity of the technique^11^, since the final sequence reads do not represent the original ctDNA populationbecause of the global amplification step during template preparation. For overcoming this problem, small collections of barcode sequences have been designed for detecting and excluding erroneous sequences. However, this approach requires each barcode sequence to be individually manufactured, and it cannot accommodate large numbers of sequences.

To achieve accurate ctDNA NGS analysis, we recently established a high-fidelity target-sequencing system of individual molecules identified in ctDNA using barcode sequences, which was named the non-overlapping integrated reads (NOIR) sequencing system^11,14^. Analysis using this system consists of the following steps. First, a novel target sequencing method is used for adding barcode sequences via adapter ligation. This method uses linear amplification for eliminating errors introduced in the barcode sequence during the early cycles of PCR. Second, erroneous barcode tags are monitored and removed^11^. This process involves the identification of individual molecules that have been sequenced and that have undergone absolute quantitation of the number of mutations. Using this method, we succeeded in evaluating the response to anticancer drugs by sequencing ctDNA in patients with advanced pancreatic cancer^15^.

Furthermore, we attempted to measure the fragment length of ctDNA accurately by using the NOIR sequencing. Tumor-derived ctDNA, which undergoes fragmentation is shorter than circulating cell-free DNA^16,17^. ctDNA fragmentation has been reported to be associated with cancer prognosis and malignancy^15,18–20^ , and examining fragmented ctDNA has been suggested to be effective in detecting tumor-derived mutations^21,22^. Observations regarding differences in fragment length size and, to a lesser extent, periodicity were translated to a patient with widely metastatic melanoma, where the principal peak associated with the *BRAF* V600E mutation was shorter than the wild-type distribution. Similar differences in fragment size were observed in patients with lung cancer harboring common *EGFR* major activating mutations^23,24^. Tumor-derived ctDNA is believed to be released during apoptosis and necrosis of tumor cells^25,26^, and nucleosome-bound cancer DNA is more susceptible to endonucleases in the process, increasing its likelihood of fragmentation^27^. ctDNAs of 100 bp or less are classified as ultrashort-fragment ctDNAs^28^, and ctDNAs with hot point mutations are often classified in this category^24^. We investigated whether the ctDNA fragment length predicts the effect of EGFR-tyrosine kinase inhibitor (TKI).

In the present study, we analyzed ctDNA using the NOIR sequencing system in patients with non-small cell lung cancer (NSCLC) harboring *EGFR* T790M mutation to evaluate the therapeutic effect of osimertinib and behavior of tumor subclones during osimertinib treatment.

## Results

### Patient characteristics

From January to December 2018, 34 patients with *EGFR*-mutated NSCLC harboring the *EGFR* T790M mutation acquired after the first EGFR-TKI treatment were treated with osimertinib monotherapy. The clinical characteristics of the patients are summarized in Table 1. The median age of these patients was 66 years (IQR, 39–83 years); 27 patients (79.4%) were female, 22 patients (64.7%) were never smokers, and 17 patients (50%) had brain metastases at the time of osimertinib administration. The *EGFR* mutation profiles were as follows: Ex.19 del (58.8%), Ex.21 L858R (41.2%), and resistance mutation T790M (100.0%). Among the patients, 10 (29.4%) received osimertinib as second-line treatment; 11 (32.4%), as third-line treatment; and 13 (38.2%) after the fourth line of treatment. Median progression free survival (PFS) with osimertinib treatment was 6.6 months (95% confidence interval [CI]: 5.1–12.9) in all patients, 7.1 months (95% CI: 5.1–15.3) in patients with the Ex.19 deletion, and 6.0 months (95% CI: 2.8–15.5) in those with the Ex.21 L858R mutation. No significant difference in median PFS was observed among the major *EGFR* mutations.

**Table 1:** Patients’ characteristics.

	Enrolled patients	(%)
	(n = 34)	
**Age-median, [range]**		
Median	66 [39–83]	
**Sex-no. (%)**		
Male	7	(20.6)
Female	27	(79.4)
**Smoking**		
Never	22	(64.7)
Ex- or Current	12	(35.3)
**PS**		
0	14	(41.2)
1	16	(47.1)
2	4	(11.7)
**Histology-no. (%)**		
Adenocarcinoma	33	(97.1)
Adenosquamous	1	(2.9)
**Stage-n. (%)**		
IVA	7	(20.6)
IVB	27	(79.4)
Brain metastases at osimertinib	17	(50.0)
**EGFR mutation**		
Ex. 19 deletion	20	(58.8)
Ex.21 L858R	14	(41.2)
**Osimertinib treatment line**		
2	10	(29.4)
3	11	(32.4)
4≧	13	(38.2)

### Fragmentation of *EGFR*-mutated ctDNA predicts osimertinib efficacy

**Figure 1:**
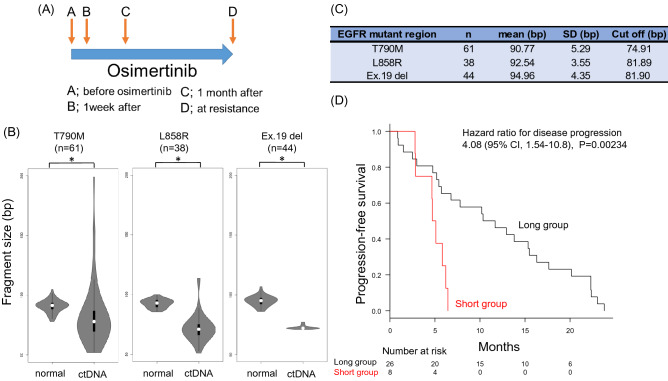
(**A**) Schematic representation of the research protocol. Peripheral blood was collected before administration of osimertinib (point A), one week after administration (point B), one month after administration (point C), and when osimertinib resistance was identified (point D). (**B**) Violin plot of *EGFR* wild-type and mutated ctDNA fragmentations. White circles (〇) indicate median levels. The plots represent individual EGFR-mutated alleles and the respective number of cases; *p < 0.05. (**C**) The median, standard deviation (SD), and cut-off values of each fragment size of *EGFR* T790M, L858R, and Ex.19 deletion alleles. (**D**) Kaplan–Meier plot of PFS for osimertinib treatment. Comparison of PFS for *EGFR*-mutated patients with the shorter *EGFR*-mutated allele fraction (red line) vs. others (black line); HR for the long group: 4.08 (95% CI, 1.54–10.8; *p* < 0.05).

A total of 34 peripheral blood samples were collected from each point A to C, and 30 samples were collected from point D (Fig. 1A). A total of 132 samples were successfully collected, and samples were collected in 30 cases through points A to D. Median cfDNA concentration (ng/µL) at point A was 0.6 ng/µL (IQR, 0.3–4.2 ng/µL), and the median total DNA content was 115 ng (IQR, 67–836 ng). The median optical density (OD) 260/280 of cfDNA was 1.79 (IQR, 1.63–1.92). Fragment size was examined for each ctDNA harboring *EGFR* major mutation among 132 samples. The fragment sizes of ctDNA for Ex.19 del (n = 44), Ex.20 T790M (n = 61), and Ex.21 L858R (n = 38) were significantly smaller than that of their corresponding normal *EGFR* coding fragments (Fig. 1B). The mean size of each ctDNA fragment is shown in Fig. 1C. Notably, following osimertinib treatment, *EGFR*-mutated patients with a shorter *EGFR*-mutated ctDNA fragment (4.9 months; 95% CI: 2.8–13.8) showed a shorter PFS than patients with a longer EGFR-mutated ctDNA fragment (11.0 months; 95% CI: 5.5–15.5; short vs. long; P = 0.00234) (Fig. 1D).

### Time of disappearance of *EGFR*-mutated ctDNA is not associated with osimertinib efficacy

**Figure 2:**
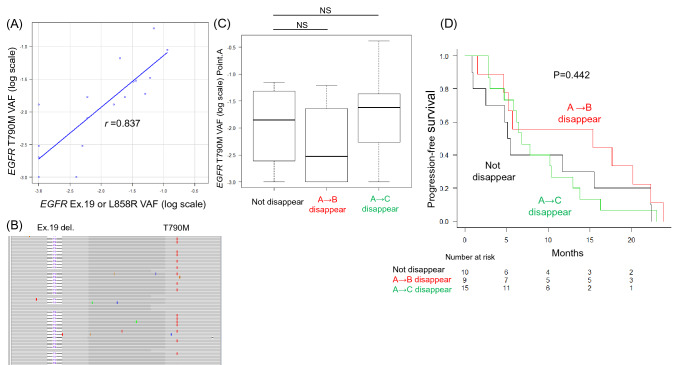
(**A**) A scatterplot graph and correlation analysis (r = 0.837) results for VAF of the *EGFR* Ex.19 deletion or L858R mutations (X axis) and the *EGFR* T790M mutations (Y axis) (**B**) The cis–trans configuration between the activating and resistance mutations was analyzed by target RNA-Seq analysis of tissue biopsy specimens. This figure shows an example of a cis pattern of Ex.19 deletion and T790M visualized by the IGV genome browser. The region bridging Ex.19 and Ex.20 of *EGFR* was amplified by RT-PCR and the amplicon was sequenced by NGS. Most sequencing reads with a 15-bp Ex.19 deletion (represented by the blue line and number) harbor T790M mutations on the same RNA molecule. (**C**) Comparison of the VAF of *EGFR* T790M at point A between the no disappearance, A → B disappearance, and A → C disappearance groups. NS; no significance. (**D**) Kaplan–Meier plot of PFS for osimertinib treatment. Comparison of PFS between the no disappearance, A → B disappearance, and A → C disappearance groups (*p* = 0.442).

A positive correlation was found between Ex.19 del, L858R VAF, and T790M VAF at point A (*r* = 0.837) (Fig. 2A). In 19 cases (55.9%) in which primary tissue specimens were obtained at point A, we examined whether the T790M location was *cis* or *trans* to Ex.19 del or L858R; 18 (94.7%) presented the T790M mutation in the *cis* position (Fig. 2B). In one case, the T790M mutation was in the *trans* position. All 34 cases were classified according to the time of disappearance of both the Ex.19 or L858R and T790M mutation alleles. Cases that showed disappearance at point B were classified into the A → B disappearance group (n = 9); those that showed disappearance at point C were classified into the A → C disappearance group (n = 15); and the remaining cases were classified into the no disappearance group (n = 10). The three groups showed no significant difference in the T790M VAF at point A (*p* = 0.379) (Fig. 2C) and no significant difference in PFS with osimertinib treatment (*p* = 0.442) (Fig. 2D).

### Evolution of the resistance mutational pattern is associated with osimertinib efficacy

**Figure 3:**
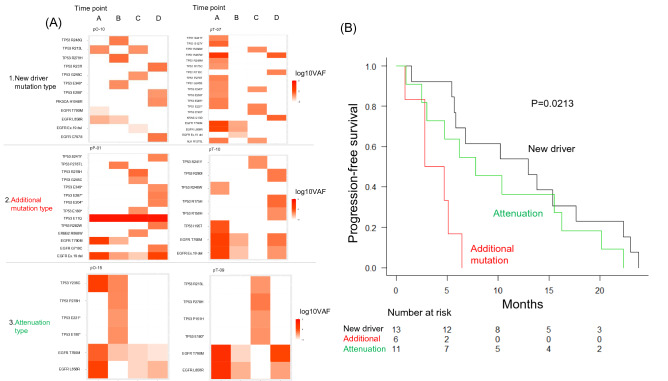
(**A**) Representative heat maps of clonal evolution types (new driver mutation type, additional mutation type, and attenuation type). The Y axis represents each mutation, and the X axis represents VAF from point A to D. The percentage of VAF is expressed in alongside heat map tones. In the new driver mutation type, *EGFR* mutation disappears and a new driver mutation appears in point D. In the additional mutation type, additional driver mutations appear in addition to *EGFR* mutation. In the attenuation pattern, driver mutation only of *EGFR* mutation is confirmed even in point D. (**B**) Kaplan–Meier plot of PFS for osimertinib treatment. Comparison of PFS between the new driver mutation type, additional mutation type, and attenuation type (*p* < 0.05).

For the evaluations of the mutation profile at point D, 30 cases that could be analyzed were classified into the following three groups. In the first group, *EGFR* Ex.19, L858R, and T790M mutations disappeared and a new mutation appeared at oncogenic driver genes other than *EGFR* (new driver mutation group; n = 13); in the second group, a new oncogenic driver mutation was added to the existing *EGFR* mutation (additional mutation group; n = 6); and in the third group, only the existing *EGFR* mutations were detected (attenuation group; n = 11). Two representative examples of each of these three groups are shown in Fig. 3A. The additional mutation group showed a significantly shorter PFS (3.8 months; 95% CI: 0.9-NA) among the three groups (*p* = 0.00221) (Fig. 3B).

**Figure 4:**
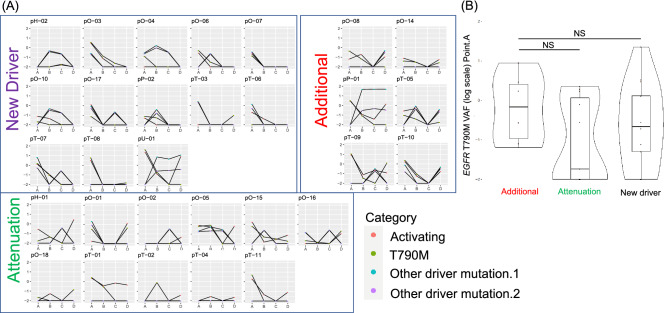
(**A**) Graphical plot of the variation of VAF for *EGFR* major mutation, T790M mutation, and other driver mutations for the 30 cases, for which samples were collected from points A to D. It is divided into three types according to the evolutional pattern: new driver, additional driver, and attenuation patterns. (**B**) Comparison of the VAF of EGFR T790M at Point A between the new driver, additional driver, and attenuation patterns. NS; no significance.

Upon plotting the transition of the VAF of the activating mutation of *EGFR* Ex.19 del or L858R, T790M mutation, and new driver mutations in 30 cases (Fig. 4A), it was suggested that in the additional mutation group, the clone at point.A disappeared once and a new resistant clone harboring *EGFR* mutation emerged at point.D. In the new driver mutation group, a new resistant clone without *EGFR* mutation emerged after all existing clones disappeared. T790M VAF at point.A was not significantly different (Fig. 4B); however, a trend toward higher VAF was observed in the additional mutation group.

### NGS concordance of tissue and plasma samples and co-mutation profile

**Figure 5:**
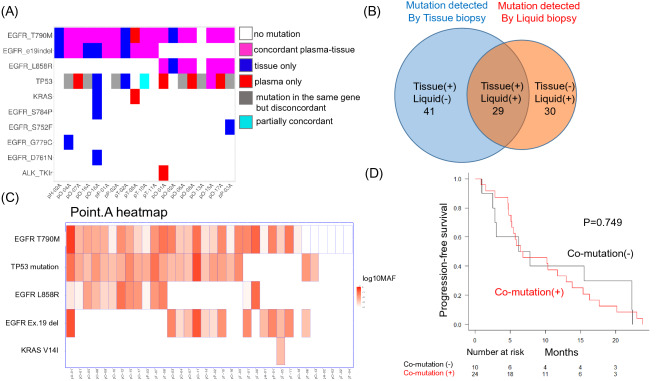
(**A**) Oncogene mutation concordance chart for mutated genes across patients analyzed by tissue–liquid-matched pairs. A total of 19 patients were tested using both NOIR-SS NGS assays. The detected mutations were colored according to the concordance status. (**B**) Mutation fractions of the shared versus tissue- or liquid-only mutations. (**C**) Heatmap of ctDNA mutations detected by liquid biopsy at point A. The detected mutations were colored based on the basis of their log_10_ VAF values. The mutation with the highest VAF value was selected in this heatmap when multiple mutations were detected for TP53. (**D**) Kaplan–Meier plot of PFS for osimertinib treatment. Comparison of PFS between co-mutation(−) and co-mutation( +) groups. *p*-value = 0.749.

A total of 19 cases for which tissue samples at point A were available were analyzed using NGS with the NOIR-SS panel for assessing the concordance rate with plasma samples. The results are presented as a mutation heat map in Fig. 5A. A total of 100 mutations were detected in the plasma samples and the corresponding tumor tissue at point A. Of the 100 mutations, 29 (29%) were shared among both samples, 41 (41%) were detected only in tissue, and 30 (30%) were detected only in the plasma (Fig. 5B). The mutations detected in 16/19 (84.2%) cases were consistent only for *EGFR* major mutations^29^. On the basis of the sequencing of plasma ctDNA at point A, 24 cases were classified as co-mutation-positive (Fig. 5C). Osimertinib treatment resulted in no significant difference in PFS between the co-mutation-positive and co-mutation-negative groups (*p* = 0.749) (Fig. 5D).

### Predictive ctDNA biomarker for osimertinib efficacy

Multivariate analysis of the influence of ctDNA biomarkers on osimertinib efficacy for patients with *EGFR*-mutation demonstrated that short fragmentation of *EGFR*-mutated ctDNA was an independent predictive factor for PFS in osimertinib treatment after second-line therapy (HR, 3.76; 95% CI: 1.17–12.1) (*p* < 0.05) (Table 2).

**Table 2:** Analysis of clinical and ctDNA risk factors for PFS of osimertinib.

Variables	(%)	Univariate		Multivariate	
		HR (95% CI)	*p*	ORR (95% CI)	*p*
**EGFR major mutations**					
Ex.19 del	(58.8)	1	Reference	1	Reference
L858R	(41.2)	1.60 (0.77–3.33)	0.209	1.53 (0.64–3.67)	0.340
**EGFR-mutated ctDNA fragment**					
long	(76.5)	1	Reference	1	Reference
short	(23.5)	4.08 (1.54–10.8)	0.0047	3.76 (1.17–9.14)	0.035
**Disappearance timing**					
A → B disappear	(26.5)	1	Reference	1	Reference
Others	(73.5)	1.68 (0.75–3.77)	0.21	2.13 (0.79–5.75)	0.133
**Clonal evolution patterns**					
New driver	(43.3)	1	Reference	1	Reference
Attenuation	(36.7)	1.53 (0.66–3.56)	0.320	1.41 (0.54–3.69)	0.483
Additional	(20.0)	3.46 (2.03–5.46)	0.0152	1.16 (0.74–1.82)	0.112
**Co-mutations**					
(−)	(29.4)	1	Reference	1	Reference
( +)	(70.6)	1.07 (0.50–2.29)	0.869	0.90 (0.35–2.26)	0.815

## Discussion

The use of liquid biopsy, which mainly employs ctDNA analysis, is growing in many types of cancers, including NSCLC. Recent technological innovations in ctDNA analysis have enabled the utilization of ctDNA analysis in a manner that is compatible with clinical practice, and two commercially available plasma-based NGS assays (Guardant360 and FoundationOne Liquid CDx) have received Food and Drug Administration approval. In addition to identifying druggable oncogenic mutations, ctDNA analysis has been widely used to identify new biomarkers, assess tumor clonal evolution, and obtain information for subsequent therapies. In this study, we demonstrated that ctDNA fragmentation is a significant predictor of the response to osimertinib therapy in *EGFR*-mutated NSCLC patients with the T790M mutation. Dynamic changes in *EGFR*-mutated ctDNA and clonal evolution may also be predictive of osimertinib efficacy; further analysis is required regarding the clinical application of ctDNA.

In the present study, the mean ctDNA size of the major mutations of *EGFR*, namely, T790M, Ex.19 del, and L858R, ranged from 90.77 to 94.96 bp (Fig. 1B,C). Fragmentation of ctDNA is related to the degree of tumor cell apoptosis in the tumor microenvironment or the degree of local inflammation in the tumor^30,31^, and ultra-short ctDNA fragments may serve as surrogate markers of the degree of local tumor inflammation. The increased degree of ctDNA fragmentation suggests a high frequency of tumor cell turn over and may indicate a high tumor growth rate^32^. EGFR-TKIs may be less effective when local tumor inflammation is severe^33,34^, which may account for the lower efficacy of osimertinib in patients with ultra-short fragments (Fig. 1D). Ultra-short fragmented ctDNA cannot be detected by RT-PCR in terms of size^28^, and accurate measurement of mutated ctDNA by the NOIR sequencing system^11^ may have successfully identified biomarkers of ctDNA fragmentation. According to univariate analysis, short-fragmented ctDNA and additional driver evolution pattern were each associated with short PFS following osimertinib treatment; in contrast, multivariate analysis showed that only short-fragmented ctDNA was significantly associated with short PFS following osimertinib treatment (Table 2). Evolution patterns are manifested by the influence of osimertinib, and short-fragmented ctDNA prior to administration is independent of evolution patterns and a promising marker for predicting osimertinib efficacy.

All enrolled patients in this study were treated with osimertinib after acquiring resistance mutations in EGFR T790M following EGFR-TKI therapy. In most cases, in which the positional relation between T790M and major activating mutations could be examined using tissue samples, the T790M mutation was located in the *cis* location, and the VAF for each mutation showed a strong correlation (Fig. 2A,B). The mutant allele dynamics of T790M and the major activating mutations were expected to be identical. The dynamics of ctDNA, especially the disappearance of mutant alleles after the introduction of osimertinib, have been reported to predict long-term efficacy^35–37^. Consistent with this result, in the present study, we observed a trend toward a longer response to osimertinib in patients showing early disappearance of T790M and major activating mutation alleles; however, the difference was not significant.

In this study, osimertinib treatment resulted in a very short PFS of 6.6 months compared to the PFS of 10.1 months in the AURA3 trial^38^. This may be because, in the present study, 50% of the enrolled patients had brain metastases at the time of treatment and 70.6% of the patients had received at least two regimens prior to osimertinib. In the AURA3 trial, 96% of the patients were administered osimertinib as the second line treatment^38^; therefore, the difference in PFS between the two trials may be attributed to the difference in patient background.

In the phase 3 AURA3 trial^38^, which compared osimertinib with platinum–pemetrexed combination chemotherapy in patients with *EGFR* T790M-positive NSCLC following progression during prior EGFR-TKI treatment, analysis of the mechanisms of osimertinib resistance showed that 21% of the patients acquired additional compound *EGFR* mutations and retained the T790M mutation even after they showed osimertinib resistance^39^. In the present study, the C797S mutation, which is the most common tertiary *EGFR* mutation^38^, was detected in one patient and was found to be in the *cis* position relative to the T790M mutation (additional mutation type). In cases showing compound *EGFR* mutations, retention of the T790M mutation suggests that this additional *EGFR* mutation may be in the *cis* position. In approximately 50% of cases, the T790M mutation allele disappears in resistance, and the manifestation of new driver mutations, such as *KRAS* and MET amplification, can be recognized. Our study additionally found new driver mutations in 13/30 (43.3%) patients (Fig. 3), which is consistent with these findings. Analysis of the resistance mechanism in the AURA 3 trial and the effect of osimertinib showed that loss of the T790M mutation allele was associated with a shorter PFS in osimertinib-treated patients^40^ . In our study, the addition of a new driver mutation (additional mutation type) was associated with a shorter PFS, and the loss of the T790M mutation allele tended to result in a longer PFS than the additional mutation type. The following factors may account for these differences. First, in our study, 24/34 (70.6%) patients received post-third-line osimertinib therapy, and it is likely that the clonality in these tumors was different from that in the AURA 3 trial population, owing to the effects of drugs other than EGFR-TKIs. Second, the previous study only investigated the loss of the T790M mutation allele and did not estimate the behavior of major activating *EGFR* mutation alleles^40^. Our results suggest that clones with an additional novel driver mutation in *EGFR* T790M and major activating mutations are resistant to osimertinib and that they may be related to shorter PFS.

The serial ctDNA analysis in our study showed the emergence of *TP53* and *ERBB2* mutations in the middle (point B or C) and their disappearance in the resistant cases (point D). Under treatment pressure, a new mutant clone may appear and disappear rapidly in the tumor^41^. The PFS achieved with osimertinib treatment was the shortest in the additional mutation type (Fig. 3B). The VAF plot of *EGFR* and other mutations shows that the *EGFR* mutant clones appear to have disappeared at points.B and C (Fig. 4A); however, new *EGFR*-mutant clones harboring additional mutations appear. Unfortunately, this study cannot clarify whether these resistant clones with additional mutations existed at the start of treatment or emerged upon osimertinib administration. However, clones with multiple driver mutations have been suggested to be highly proliferative and may eliminate other clones, suggesting that they may have been generated by osimertinib administration rather than having existed prior to the start of treatment. The PFS was the longest in the new driver mutation type group. According to the VAF plots, *EGFR* mutant clones almost disappeared and clones without *EGFR* mutation appeared during resistance. Unlike the additional mutation type, the resistant clone could present as a minor clone at the start of osimertinib treatment. The PFS of the osimertinib-treated patients was longer possibly because the *EGFR* mutant clone was eliminated with osimertinib administration and it took time for the minor clone to become apparent. In the future, when evaluating the genomic profile of the primary tumors using ctDNA, the timing of blood collection and the timing of treatment change must also be considered. The timing of the appearance of new mutations, resistance formation in ctDNA, and tumor progression in images should be studied in conjunction with each other. Additiobally, on the basis of prospective studies, guidelines should be developed for ctDNA-guided treatment.

In the present study, the concordance rate of tissue- and plasma-based NGS was as low as 29/70 (41.4%) (Fig. 4A,B). In a previous report, the concordance rate of major druggable mutations between plasma- and tissue-based NGS using Guardant360 was 77.6%^29^. In this study, the concordance rate of *EGFR* major mutations was 84.2%, however, the concordance rate of *TP53* mutations was low (Fig. 4A). This study included many cases after a relatively long period of treatment, and the heterogeneity of the primary and metastatic lesions at this point may have possibly increased. Intratumoral clones are greatly affected by the effects of TKIs; in this study, such discordance was attributed to the effect of prior treatments before analysis^42,43^. In addition, no previous studies have used the same analytical panel for tissue and plasma in previous reports; however, the present study used the same panel for both. ctDNA tends to be fragmented^22,44^, and differences in the quality of DNA in comparison with the DNA extracted from tissue may have led to differences in detection rates. The concordance rate could be maintained in the case of major druggable mutations^29^; however, the concordance rate might be lower in the case of other mutations. The effect of EGFR-TKIs on patients with *EGFR*-mutation is influenced by co-mutations other than major *EGFR* mutations; however,the present study failed to confirm the effect of co-mutations on the effect of EGFR-TKIs (Fig. 5D). In this study, only a small number of patients were analyzed, and the timing of EGFR-TKI administration was inconsistent. More than 50% of the patients received late line EGFR-TKI administration (Table 1).

This study had multiple limitations. Samples were collected for only 34 cases, and sample collection from points A to D was successfully performed only in 30 cases. In addition, tissue samples at point A were successfully collected in only 19 cases, and successful comparisons with tissue samples were performed in only a few cases. The gene panel used in this analysis was a very small panel of only nine genes and was primarily aimed at searching for variations in *EGFR* and *TP53* mutations. A comprehensive panel of genes may allow identification of specific gene mutations associated with osimertinib efficacy. The timing of the introduction of osimertinib varies from the second, third, and fourth to later lines of treatment, and it is possible that the influence of drugs administered prior to the introduction of osimertinib possibly caused bias in the genomic profile investigated to predict the therapeutic effect of osimertinib, yielding results different from those reported previously. Additionally, the timing of image evaluation for the effects of osimertinib was not defined in terms of parameters other than the sample collection; thus, the findings may not provide an accurate evaluation of osimertinib efficacy. Because the findings were not adjusted for the presence or absence of brain metastases and other distant metastases, systemic conditions at the start of treatment may have influenced osimertinib efficacy.

## Conclusion

In this study, using serial plasma samples from patients harboring EGFR T790M and major activating mutations who were treated with osimertinib after second-line therapy, we analyzed the effect of osimertinib, the relationships among ctDNA biomarkers, and the changes in gene mutation profiles in ctDNA by utilizing the NOIR sequencing system. Multivariate analysis revealed that the fragment size of ctDNA with *EGFR* mutations could predict the effect of osimertinib. Since the size of ctDNA as well as the mutation profile can be used as biomarkers for predicting therapeutic effects, their use as predictors should be analyzed in a large-scale study in the future.

## Methods

All methods were carried out in accordance with relevant guidelines and regulations including the Declaration of Helsinki. The present study was a protocol-based retrospective study, and the study protocol (UMIN000028990) was approved by the ethics committees of each of the five participating institutes (Osaka International Cancer Institute, Osaka Toneyama Medical Center, Osaka Police Hospital, Osaka Habikino Medical Center and Osaka University Hospital). We enrolled NSCLC patients with presenting biopsy-proven, advanced, nonsquamous NSCLC harboring *EGFR* T790M mutations in addition to the *EGFR-*activating major mutations exon (Ex.) 19 deletion (del) or Ex.21 L858R after EGFR-TKI treatment on the basis of results of peptide nucleic acid-locked nucleic acid (PNA-LNA) real-time polymerase chain reaction^45^; exhibiting an Eastern Cooperative Oncology Group performance status of 0–2; and scheduled to receive oral osimertinib (at a dose of 80 mg once daily). Treatment continued until disease progression, the development of unacceptable adverse effects, or a request by either the patient or physician to discontinue treatment. Patients with concurrent malignancy were ineligible, except those with non-melanoma skin cancer or noninvasive cervical cancer. Patients with a history of a prior cancer other than NSCLC were included if the previous diagnosis was performed more than 2 years prior to enrollment and if the patient had no evidence of active disease. Patients had to have a life expectancy of at least 3 months. Patients with central nervous system metastases were eligible if they had been adequately treated and the neurological findings had returned to baseline. Brain metastases were screened by brain computed tomography (CT) or magnetic resonance imaging (MRI) in all patients before the initiation of treatment. Patients with a history of osimertinib use were excluded from the study. Written informed consent was obtained from each patient or their guardian.

### Specimen collection and plasma isolation

Blood samples (20 mL) were collected before starting osimertinib treatment, one week and one month after starting osimertinib treatment, and when resistance to osimertinib was recorded (Fig. 1A). Whole blood samples were collected in Streck cfDNA blood collection tubes, processed to plasma, and frozen within 96 h. Whole blood was centrifuged at 1,600 × *g* for 10 min. The plasma supernatant was isolated and centrifuged twice at 4,122 × *g* for 15 min (swinging bucket, break-off), aliquoted, and frozen at − 80 °C for future use.

### Sample preparation, quality assessment, and NOIR-SS assay design

Plasma preparation was performed as described previously^46^. Cell-free DNA was extracted from patient plasma samples by using a QIAamp circulating nucleic acid kit (QIAGEN, Valencia, CA, USA). cfDNA was concentrated using Amicon Ultra-0.5 centrifugal filters. Double-stranded DNA was quantified using the Qubit dsDNA HS Assay (Thermo Fisher Scientific, Waltham, MA, USA) on a Qubit 2.0 Fluorometer (Thermo Fisher Scientific). A molecular barcoded NGS library was constructed using the NOIR-SS method, as described previously^11^. Purified cfDNA from 9 mL of blood was used as the starting material for the NOIR-SS assay. The amplicon panel (Table S1) covering lung cancer-related genes was used for amplifying the target regions. The primers and adapters used for library construction are shown in Table S2. For each plasma sample, to avoid undesirable amplification between forward and reverse gene-specific primers, we prepared two reaction mixtures for forward and reverse gene-specific primer cocktails (Table S2) since our PCR system did not allow simultaneous use of forward and reverse primer pairs. The sequencing library was amplified by anchored PCR using a primer pair between a single anchor of a gene-specific primer and a universal primer on the sequencing adapter.

### Sequencing and data analysis

The constructed library was quantified using the Qubit dsDNA HS Assay Kit or Quant-iT PicoGreen dsDNA Assay Kit (Thermo Fisher Scientific) and loaded on an Ion 540 chip using the Ion Chef System (Thermo Fisher Scientific). Sequencing was performed using the Ion Torrent S5 XL platform. Data analysis was performed according to a previously described procedure^46^. Reads in the FASTQ format were de-multiplexed using the 5-base indices for assignment of individuals. The sequences between the 5-base indices and spacer/linker sequences were obtained and used as barcode tags (unique molecular identifiers). Reads with more than 50 bp were aligned to the target regions by using BWA-MEM^47^, whereas reads with short mapped ends (< 40 bases) were discarded. Reads with the same molecular barcode sequences were grouped together, and erroneous barcode tags were detected and removed, as described previously. Consensus sequences for reads with the same barcode were created using VarScan^48^ as described previously^11^. When more than 85% of the reads had the same base at a position, this base was selected as the consensus base.

### Variant call by molecular barcoding analysis

For variant detection, we applied a Poisson distribution model to calculate the sequencing error, as described previously^11^. When the number of base alterations in a target region is significantly higher than the average expected from the sequencing error, the change may be attributable to variant(s). The sequencing error rate was set to 10^− 5^, corresponding to the expectation of one alteration at a single nucleotide site by sequencing error in 100,000 analyzed DNA molecules. In this study, we evaluated each target site for the presence of variant(s), setting *p* = 0.01 as the threshold for variant detection. For hotspot mutations with variant-positive molecules, we evaluated each base position in the hotspot mutation at the specified threshold value estimated from anomaly detection^49^. To remove artifactual mutations due to DNA damage or somatic mutations from normal hematopoietic stem cells (clonal hematopoiesis), CV78 variant filtering^46^ was applied for selecting variants of somatic mutations using the COSMIC database version 84. Common SNP sites and error-prone sites were excluded from the analysis. We used human genome build 37 (GRCh37/hg19) as the reference genome. The mutation variant was called from only a single mutant-positive DNA molecule when the probability of the observation estimated from the Poisson distribution under the null hypothesis was less than the p-value cutoff. The minimum %VAF for the variant call depended on the total DNA fragments analyzed for the sample. On an average, 3,589 cfDNA molecules were analyzed in the NOIR-SS assay in this study, corresponding to an average limit of detection %VAF of 0.03% (1/3,589). Oncogenic mutations were defined as those appearing in addition to the main activating mutations, and mutations other than those registered as somatic mutations in COSMIC and common SNPs registered as HGVD were detected.

### ctDNA fragment size analysis

Using the anchored PCR assay of NOIR-SS, the molecular tag adapter was attached to the blunt end terminal of cell-free DNA. The cfDNA from normal cells is considered to be fragmented by endogenous endonuclease digestion of nucleosome junctions at the time of apoptotic cell death. DNA damage generates short fragments of cfDNA during library preparation. Furthermore, cell death by necrosis or pyroptosis causes disordered and random fragmentation, regardless of the nucleosome structure. Therefore, the fragment size pattern of cfDNA/ctDNA potentially contains information on the status of the tumor microenvironment, such as inflammation and the degree of abnormal cell death (necrosis/pyroptosis). The difference in cfDNA or ctDNA was discriminated according to the existence of a tumor marker sensitizing mutation (*EGFR* L858R, Ex.19 del, T790M) on the fragment DNA, and the fragment size distribution was estimated. The fragment size was estimated from the DNA length between the gene-specific primer-binding site and the terminal end of cfDNA. The 3σ method^50,51^ was used for evaluating the fragment size of the *EGFR*-mutated ctDNA. Using this method, we defined “short fragment group” as those outside the standard range of ctDNA size.

### The *EGFR* allelic cis/trans location analysis

The allelic cis/trans conformation of activating/resistance mutation pairs was assessed by target RNA sequencing using purified RNA as the starting material. First-strand cDNA was synthesized using Revertra-ace® (Toyobo), and multiplex PCR was performed using KOD Fx Neo (Toyobo). RT-PCR and sequence library construction were performed using the same assay conditions as described previously^52^. Two sets of primer pairs for amplifying activating/resistance mutation pairs were used for revealing the cis/trans configuration by analysis of the *EGFR* exon20-21 amplicon for T790M-L858R detection and the exon19-20 amplicon for Ex.19 del.-T790M detection. A total of 100 ng of input RNA was used for each reverse transcription reaction assay. Sequence libraries for the Illumina platform from these PCR products were constructed using the GenNext® NGS Library Prep Kit (Toyobo). All procedures were performed according to the manufacturer's instructions. To determine the cis/trans configuration, approximately 5 K depth of reads in average was obtained for each amplicon and the minimum cutoff for mutation call was set to 10 positive reads per amplicon. The sample was judged as harboring compound mutations by cis conformation when the activating-resistance mutation pair was called on the single identical read, and it was otherwise judged as showing a trans conformation.

### Statistical analysis

Efficacy assessments were performed every 8 weeks until the investigator’s detected RECIST-defined disease progression. Imaging assessments included chest, abdomen, and brain CT or MRI. A patient was considered to have stable disease if this response was confirmed and sustained for 8 weeks. PFS was defined as the time from enrollment to the date of confirmation of progressive disease or the date of death from any cause. For analyzing PFS, times to events were estimated using the Kaplan–Meier method and compared using the log-rank test. Hazard ratios (HRs) for PFS were determined using a univariate Cox proportional hazards model. Cox proportional hazard models were used for evaluating several patient factors. To construct the multivariate model, we selected the most relevant PFS-related factors identified in the univariate analysis. All statistical analyses were performed using the EZR software (Saitama Medical Center, Jichi Medical University, Saitama, Japan)^53^. Statistical significance was set at *p* < 0.05.

### Ethical approval

The present study was a protocol-based retrospective study, and the study protocol (UMIN000028990) was approved by the ethics committees of each participating institute. Informed consents were obtained from the all cases who were enrolled in the study.

## Supplementary Information

Supplementary Information.

## Data Availability

The datasets generated during and/or analyzed during the current study are available in the DDBJ Sequence Read Archive repository. DRA submission ID is PSUB016571.
